# Understanding leisure‐time physical activity: Voices of people with MS who have moderate‐to‐severe disability and their family caregivers

**DOI:** 10.1111/hex.12600

**Published:** 2017-07-19

**Authors:** Afolasade Fakolade, Julie Lamarre, Amy Latimer‐Cheung, Trisha Parsons, Sarah A. Morrow, Marcia Finlayson

**Affiliations:** ^1^ School of Rehabilitation Therapy Queen's University Kingston ON Canada; ^2^ School of Kinesiology and Health Studies Queen's University Kingston ON Canada; ^3^ Multiple Sclerosis Clinic University Hospital—London Health Services London ON Canada

**Keywords:** caregivers, moderate‐to‐severe disability, multiple sclerosis, physical activity

## Abstract

**Background:**

Physical activity (PA) is beneficial for all people, yet people affected by multiple sclerosis (MS) find regular PA challenging. These people may include individuals with the disease who have moderate‐to‐severe disability and their family caregivers. For researchers and clinicians to effectively promote PA among caregiver/care‐recipient dyads with moderate‐to‐severe MS, a comprehensive understanding of the shared PA experiences of these dyads would be beneficial.

**Objective:**

We explored shared experiences of caregiver/care‐recipient dyads affected by moderate‐to‐severe MS about PA and directions for intervention.

**Methods:**

Six focus groups with 23 people with moderate‐to‐severe MS and 12 family caregivers were conducted. Data were analysed using a constant comparative approach.

**Results:**

Three major themes emerged as follows: (i) PA is a continuum, (ii) cycle of disengagement and (iii) cycle of adjustment. The first theme captured the dyads understanding that PA falls along a continuum ranging from highly structured to unstructured activities. Cycle of disengagement captured the experiences of dyads engaging in little or no PA. These dyads perceived internal and external issues as drivers of the cycle of disengagement, while availability of supportive programmes and services or people helped the dyads to break out of the cycle. When the cycle of disengagement was broken, the dyads described moving towards the cycle of adjustment, where they were able to learn skills and take action to incorporate PA into daily routines.

**Conclusion:**

This research highlights the need to adopt an integrative approach that acknowledges the caregiver/care‐recipient dyad with moderate‐to‐severe MS as a focus for PA intervention.

## 1 INTRODUCTION

The progressive nature of multiple sclerosis (MS) and associated motor, cognitive and psychological symptoms mean that people with this condition may become increasingly disabled as the disease progresses.^1^, ^2^, ^3^, ^4^ Consequently, many people with MS (PwMS), especially those individuals with moderate‐to‐severe disability (ie, significant walking limitations that require support for gait PDDS 3‐7 or EDSS ≥6), often need emotional, physical and instrumental support to manage associated life roles and maintain independence.^5^, ^6^ Providing this on‐going support falls primarily on the family caregivers^7^, ^8^ who may spend up to 10 hours per day for caregiving activities.^9^, ^10^ Together this evidence suggests that the impact of MS does not rest solely on the individual with disease and that PwMS and their family caregivers often need to adapt to its presence as an interdependent caregiver/care‐recipient dyad.^11^ The definition of caregiver/care‐recipient dyad adopted for this study is as follows: *the reciprocal partnership of two or more persons who enact caring roles towards one another. This partnership may include the individual with a disease and a close friend or relative usually a spouse, adult child, sibling, unmarried partner, or parent*.^12^, ^13^

Researchers have demonstrated that regular participation in physical activity (PA) has beneficial effects on all aspects of health and quality of life for all people including PwMS.^14^, ^15^, ^16^ Yet recent estimates suggest that approximately 60% of PwMS are physically inactive (ie, fail to meet public health guidelines of ≥30 min/d of moderate‐to‐vigorous intensity activity) compared with 23% of the general population.^1^ Furthermore, people with progressive subtypes of MS have lower PA levels compared to those who have a relapsing clinical course,^17^ suggesting that a higher disability level is associated with physical inactivity.^18^ With increasing caregiving responsibilities, family caregivers of people with moderate‐to‐severe MS‐related disability may have limited time and opportunity to participate in PA.^19^, ^20^

To date, PA promotion interventions in the MS literature have been individually oriented and focused primarily on individuals with mild‐to‐moderate MS‐disability.^17^, ^21^, ^22^, ^23^, ^24^ However, the developmental‐contextual model of coping with chronic disease suggests that the disease has an impact on both caregivers and care‐recipients and that dyads who engage in collaborative activities such as PA are more likely to experience better adjustment.^25^ Furthermore, research in other chronic disease contexts (eg, cancer and dementia) has demonstrated that a dyadic approach to PA improves several health‐related outcomes for the dyad including muscle strength, physical function and psychological health.^26^, ^27^, ^28^, ^29^, ^30^ Collectively, this work highlights the importance of targeting the dyad as an equal and interactive unit and suggests that PA promotion interventions that are mutually beneficial to the needs of PwMS and caregivers are warranted.

For researchers and clinicians to effectively promote PA among caregiver/care‐recipient dyads with moderate‐to‐severe MS‐related disability, a comprehensive understanding of the shared PA experiences of these dyads would be beneficial. Qualitative data are extremely valuable for providing insights to guide the development and implementation of appropriate interventions.^31^, ^32^ However, the majority of qualitative studies on PA among PwMS do so from the perspective of those with mild disability, with little consideration given to the experiences of those with moderate‐to‐severe disability.^33^, ^34^ Furthermore, no study has documented shared perspectives of caregiver/care‐recipient dyads affected by MS about PA. In response, this qualitative study was undertaken to explore shared perceptions of caregiver/care‐recipient dyads affected by moderate‐to‐severe MS‐related disability about PA. A secondary objective was to identify directions for intervention among these groups in order to inform the development of an intervention that supports their joint engagement in PA. The main question that guided our research was as follows: What are the shared perspectives of caregiver/care‐recipient dyads with moderate‐to‐severe MS‐related disability about PA?

## 2 METHODS

### 2.1 Study design

We chose a qualitative, cross‐sectional, descriptive study design to answer our research question.^35^ A focus group method was used, as it facilitates interactive discussion and exchange of ideas among participants.^36^, ^37^, ^38^ Such interactive focus group discussions are reported to provide deep insight into multiple views on health behaviours.^39^

### 2.2 Setting and participant recruitment

Focus group participants were recruited from three communities with populations ranging from 39 000 to 474 800 located within a single Canadian province. Potential participants were informed of the study through (i) a recruitment phone call to individuals who had participated in a previous study conducted by investigators in our research team and consented to being contacted for additional research projects, (ii) newspaper advertisements and (iii) information flyers distributed through local MS clinics and MS Society chapters.

The principal investigator (PI) or a trained research assistant (RA) conducted a telephone screening using a pre‐defined script developed for this study. During the phone screening, we asked the primary contact person (ie, either the PwMS or caregiver) to provide contact details of a possible partner for the focus group. Once provided, the PI or RA made a separate phone call to the partner to screen for eligibility. Potential participants were eligible to take part in the study if they met the study's eligibility criteria in Table 1.

**Table 1:** Eligibility criteria for people with MS and family caregivers

Inclusion criteria	Exclusion criteria
1. ≥18 y old^a^	1. Inability to tolerate a 90 min discussion completed entirely in English^a^
2. Self‐reported diagnosis of MS^b^	2. Unable or unwilling to attend one focus group and arrange own transportation^a^
3. A PDDS score between 3 (moderate disability) and 6 (bilateral support required)^b^	
4. Providing at least 45 min/d of support to a person with MS who has a PDDS score between 3 and 6^c^	

a Criterion applies to both people with MS and family caregivers.

b Criterion applies only to people with MS.

c Criterion applies only to family caregivers.

PDDS, Patient Determined Disease Steps; MS, multiple sclerosis. Support—any emotional, physical or instrumental help offered to a person with MS.

### 2.3 Participants

**Figure 1:**
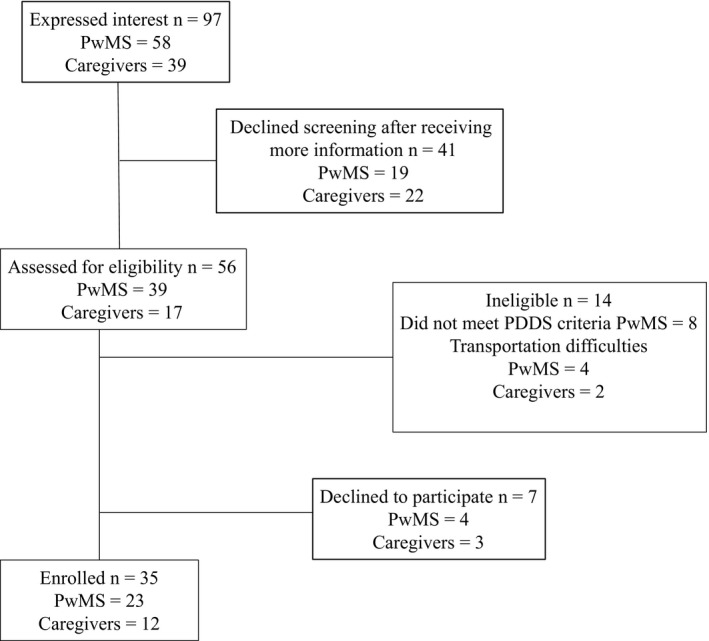
Flow chart of study recruitment and enrolment process

A total of 97 individuals (58 PwMS and 39 caregivers) expressed interest in the study. Nineteen PwMS and 22 caregivers decided not to be screened after receiving more information about the study and what was involved. After completing the screening process, nine PwMS and five caregivers were deemed ineligible (due to disease severity or transportation difficulties). Another seven PwMS were eligible but chose not to participate because the group timing was inconvenient. Thirty‐five eligible participants comprising of 23 people with moderate‐to‐severe MS‐related disability and 12 caregivers returned a copy of the signed consent materials, and these individuals were enrolled in the study. A flow chart summarizing the recruitment and enrolment process is presented in Figure 1.

### 2.4 Procedure

The focus groups were conducted between April and August 2015. Participants were assigned to groups based on practical reasons (ie, timing and locations where participants were recruited) in order to make participation as straightforward as possible. Each focus group was composed of a mix of dyads and single individuals. These single individuals were PwMS whose caregivers did not attend the groups.

Before each focus group, participants completed self‐reported questionnaires. The PwMS questionnaire captured information on the participants’ demographic (age, gender, marital status, education and current employment status) and clinical (type of MS, disease severity, years since diagnosis and impact of MS on walking) characteristics. The caregiver questionnaire captured information on the participants’ demographic characteristics (age, gender, marital status, relationship with PwMS, living arrangement, education and current employment status) and general caregiving information (years of providing support).

The PI facilitated all the focus groups, after receiving training from the senior author (MF). One additional member of the research team took notes, noted relevant non‐verbal communication, assisted with logistics and oversaw the audio recording during the groups. A semistructured discussion guide was used (Data S1). All the focus groups were digitally recorded and professionally transcribed.

### 2.5 Data management and analysis

Descriptive statistics were used to report the sample characteristics. Focus group data were analysed using a constant comparative approach.^40^ This approach was chosen to enable us to explore areas where the views of each partner converged or diverged in order to produce a shared understanding of the dyad experience of PA. The PI reviewed the transcripts against the digital recordings for accuracy and replaced participants’ names with pseudonyms. The PI and RA independently coded the transcripts after multiple readings. The coding process began with making notes in the margins of the transcripts about the participants’ comments and what was interesting about them. The next step was to develop an initial list of codes by identifying concepts embedded within the data. During this process, the two researchers compared and discussed their codes until they reached consensus. When disagreements were encountered, the senior author provided guidance. Once all the initial codes were identified, the two researchers met with the senior author to review and refine the codes, as well as develop a coding framework.

The search for themes involved condensing the codes into descriptive categories that offered a conceptualization of the shared dyad experience of PA. Another meeting with the senior author was held, in which a hierarchy of overarching themes and subthemes was discussed. This process was complemented by the development of a thematic map, which provided an overall conceptualization of the patterns within the data and relationships between them. Different iterations of the thematic map were discussed and revised with the senior author to ensure the patterns and suggested relationships represented on the map were consistent with the agreed interpretation of the data. Exemplar quotations were extracted from the transcripts to provide a basis for understanding the themes and their unifying properties. The final themes, subthemes and definitions are provided in Table 2. ATLAS.ti v7 software (Scientific Software Development GmbH, Berlin Germany) was used to facilitate data management and analysis.

**Table 2:** Themes, subthemes and definitions generated through the analysis process

Themes and subthemes		Definition
PA is a continuum		Statements describing the range of structured and unstructured activities that PwMS and their family caregivers consider PA
Cycle of disengagement	Awareness of limitations	Statements that reflect personal awareness or insights about how issues with body structure and function limit the type of activities PwMS and their caregivers engage in or how to go about engaging in such activities.
	Mourning loss	Statements where PwMS or family caregivers talk about missing or wishing they could still do activities they used to enjoy but can no longer do because of the presence of MS
	Drivers of the cycle of disengagement	Statements about factors that make it difficult for PwMS and their family caregivers to break out of the cycle of disengagement
	Inhibitors of the cycle of disengagement	Statements about factors that enable PwMS and their family caregivers to break out of the cycle of disengagement
Cycle of adjustment	Acceptance	Statements about coming to terms with the disease and accepting their new identity
	Innovation and modification	Statements reflecting how PwMS continues to work independently or together with their family caregivers to find new options or modify previously enjoyed activities

PwMS, people with MS; PA, Physical activity; MS, multiple sclerosis.

### 2.6 Trustworthiness

The strategies recommended by Lincoln and Guba^41^ were used to ensure rigour. Dependability and confirmability were achieved using an audit trail to connect the raw data and codes with themes and subthemes. To enhance credibility, two researchers independently analysed the data, discussed and compared findings and consulted with the senior author in case of a disagreement. Each participant was mailed a summary of his/her individual focus group approximately 2 weeks after the group for member‐checking. Each participant was asked to comment on the summaries if inaccuracies were detected. Participants were given the option to decline if they did not wish to be part of this procedure. Of the 35 participants, two declined to participate because of vacation plans, 21 responded and expressed no concerns with the summaries and 12 did not return the feedback form despite three follow‐up attempts. Transferability was achieved by presenting sufficient contextual information and raw data in this paper to allow readers to evaluate the themes and to decide whether they can be applied to their own situations.

## 3 RESULTS

### 3.1 Focus groups

In total, there were six focus groups and each group lasted an average of 93 minutes (SD=17.3; range=59‐107 minutes).

### 3.2 Participants

Tables 3 and 4 display the demographic and clinical characteristics of the 35 participants. The sample was largely female (n=22). PwMS were aged between 37 and 71 years. Family caregivers were aged between 38 and 79 years.

**Table 3:** Characteristics of people with MS who participated in the focus groups

People with MS (n=23)	Mean (SD)	Min	Max
Age	54.6(9.8)	37	71
PDDS	4.7(0.9)	3	7
Disease duration (in years)	14.7(9)	1	31
MSWS–12 score	68.4(16.7)	38	98
	n	%	
Gender			
Male	7	30.4	
Female	16	69.6	
MS type			
Primary progressive	5	21.7	
Secondary progressive	2	8.7	
Relapsing‐remitting	11	47.8	
Progressive relapsing	1	4.3	
Unknown	4	17.4	
Employment status			
Unemployed (unable to work—disability)	17	73.9	
Retired	6	26.1	
Marital status and living arrangements			
Married; living with spouse	10	43.5	
Married; living with spouse and children	6	26.1	
Single; living with parents	1	4.3	
Single; living alone	1	4.3	
Single; living with siblings	1	4.3	
Separated; living alone	4	17.4	

MSWS‐12, Multiple Sclerosis Walking Scale; MS, multiple sclerosis; PDDS, Patient Determined Disease Steps.

**Table 4:** Characteristics of family caregivers who took part in the focus groups

Family caregivers (n=12)	Mean (SD)	Min	Max
Age	57(13.8)	38	79
No. of years of providing assistance to person with MS	10.9(7)	2	20
	n	%	
Gender			
Male	6	50	
Female	6	50	
Level of education			
High school	2	16.7	
Technical/trade school	1	8.3	
College	7	58.3	
Bachelor's degree	2	16.7	
Employment status			
Unemployed (unable to work—disability)	1	8.3	
Retired	6	50	
Homemaker; does not work outside the home	1	8.3	
Part time (20‐39 h/wk)	1	8.3	
Full time (40 or more h/wk)	2	16.7	
Not working (full time caregiver)	1	8.3	
Relationship to person with MS			
Spouse	8	66.7	
Non‐spouse	4	33.3	

MS, multiple sclerosis.

### 3.3 Themes

**Figure 2:**
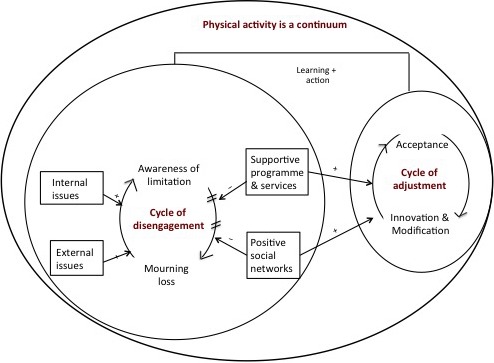
Graphic depiction of study themes and the relationship between themes

The experiences shared by the participants were captured in three related themes: “PA is a continuum,” “cycle of disengagement” and “cycle of adjustment” (Figure 2). Overall, the participants’ experiences of PA as a structured–unstructured continuum were consistent across the focus groups. The cycle of disengagement emerged from statements made by participants engaging in little or no PA. A combination of internal and external issues kept them locked in this cycle. These participants were able to engage in PA when they entered the cycle of adjustment, which occurred over a period of time. Availability and usage of adequate support systems helped them to break out of the cycle of disengagement and enter the cycle of adjustment. In the discussion that follows, the themes and subthemes are described with quotes that illuminate their meanings. At the beginning of each quote, a pseudonym is included with PwMS or CG used to denote a quote from a participant with MS or caregiver, respectively.

#### 3.3.1 Overarching theme: PA is a continuum

To orient the participants to the phenomenon of interest, we asked them to describe what PA meant to them. The main theme that emerged from the analysis of the focus groups was that PA falls along a continuum ranging from highly structured to unstructured activities. This theme was captured in the stories shared by both caregivers and care‐recipients. We heard some dyads describe PA as any structured, planned and supervised activity that is performed within the boundaries of their physical (dis)abilities. These dyads shared examples of aerobic and strength training activities performed in exercise facilities and under the supervision of professionals who have MS‐specific knowledge. This conceptualization of PA was most apparent in stories shared by older dyads residing in larger communities with considerably more access to facilities and services such as subsidized gym memberships.

Jason (PwMS) shared:

When I think of it [PA], I think of things like the adapted aquatic classes…what comes to mind are all the kinds of exercises I am able to do at the [name of facility] they also have a heated pool which is great…

Other dyads conceptualized PA in a fairly fluid and unstructured manner. These dyads described PA as any activity accumulated through leisure, everyday occupations and household activities (such as gardening), as well as actions taken while pursuing other goals (such as walking to work). This conceptualization of PA was mostly apparent in the stories shared by dyads affected by a more severe MS‐related disability.

Cody (CG) commented on accumulating PA through everyday household activities.

I think of gardening and cutting the grass. Even moving around the house as a caregiver. I find myself doing a lot more stairs, a little more in the garden, a little more outside. And a little more of just the day‐to‐day function around the house.

There were also examples of participants who conceptualized PA as a combination of structured and unstructured activities. This conceptualization was more apparent among PwMS, who discussed that at times (eg, during a severe relapse), they had to “take a break from the routine” [going to the gym] and move from the structured towards the unstructured end of the continuum “while recovering from the relapse” (Pat, PwMS).

Despite the wide range of possible activities that were described in the continuum, some dyads reported participating in little or no PA. During the discussions, these same dyads expressed frustration and sadness at their current level of PA. PwMS talked about their daily physical and psychological struggles when trying to be active. Family caregivers, especially those providing care to more disabled individuals, discussed the need to become experts at providing adequate care and safety for their care‐recipients during PA. In addition, these caregivers had to balance the caregiving activities while ensuring that they also were able to participate in and benefit from PA. Although these dyads expressed the desire to stay as active as possible, coping with these challenges and having to balance several dimensions of caregiving often resulted in them being unable to engage in PA. The experiences shared by these dyads were captured in the theme: cycle of disengagement.

#### 3.3.2 Theme 2: Cycle of disengagement

This theme captured the reflections of the dyads on their inability to engage in adequate amounts of PA because of issues related to physical (dis)abilities, loss of independence and the unpredictable changes associated with dealing with MS. Although this theme was consistent across focus groups, it was expressed more clearly among dyads affected by a more severe MS‐related disability. Within this theme, two subthemes emerged during analysis: *awareness of limitations* and *mourning loss*.

##### Awareness of limitations

PwMS made statements reflecting personal awareness and insights about how bodily issues limit to some extent the type of activities they engage in, as well as how they go about engaging in those activities. Caregivers also described how the limitations experienced by their care‐recipients restricted their own daily routines and participation of the entire family in PA. These same caregivers expressed the need to be constantly vigilant, monitoring the status of their care‐recipient and using it as a determinant of daily activity choices.

Kim (PwMS) commented:

I love to sew and I use a sewing machine. And my right leg is affected and it's weak, and the coordination of it. And so I have to really put a lot more energy into sitting down and sewing. I'm going to have to learn to do it with my left foot so that it's a little bit smoother. But that is another thing that it's a leisure activity for me that I always enjoyed doing that's just a little bit more difficult now because of MS

Bob (CG) also commented:

Just the speed of walking is an adjustment for me as well. I'm a fairly quick walker. When we're together, we do not walk my speed, we walk hers and sometimes it's aggravating because it's just so stinking slow… But as I'm walking, I'm trying to gauge her to see how far she's going to go and how the walking pattern is. If she's stumbling as we're walking out of the house, we're not going far… I don't know from which day to which day what it's going to be. It's just pay as you go…

##### Mourning loss

PwMS described a sense of frustration when comparing previous and current abilities. Caregivers of these individuals also expressed sadness at losing the freedom and independence to engage in activities they previously enjoyed because of the added responsibilities of caregiving.

Tina (PwMS) commented:

…To me when I think of it [PA], it's such a loss because it's more I think about what I used to do and what I would like to do…I used to dance and I used to teach fitness. I'm still grieving that loss of not being able to do that…

Tim (CG) also shared this experience:

There are chores that need to be done around the house and things that she [PwMS] used to do before that I have now taken over and that's offset some things that I used to enjoy. I used to go out for walks and hikes but I'm now sticking around a little bit more to do more around the house

##### Drivers of the cycle of disengagement

As the dyads constructed their PA experiences, a set of drivers emerged as influencing the cycle of disengagement. These drivers constantly alerted participants to their poor functional status (eg, reduced mobility) when attempting to be physically active. For some PwMS, external issues such as lack of supportive resources in the home and community, and negative feedback from others resulted in the decision not to participate in PA.

Nicole (PwMS) shared this experience about limitations with resources in the community:

…even in the community, an exercise class, they're leading for like, you know, Joe Athlete. And it's like hey, you know, don't push people that can't do it. I mean yes, push them a little bit but don't make them feel degraded because they can't keep up. So I felt…I think they made me feel that I was sort of like taking up someone else's time who needed it more than I did. And so I just quit

Eva (CG) reiterated this lack of supportive community resources:

I don't see a lot of support here. I know even with Nancy [PwMS] now they have called and they've come over and they've talked to her. But to actually recommend places to go for PA or things to do, I haven't seen that. And that's in 17 years

Cathy (PwMS) shared her experience of receiving negative feedback from others:

My daughter told me to stop walking with them basically because it's embarrassing when I fall. And she's 6…I mean it's like that because I walked with them this one particular day, and you know, I mis‐stepped, just kind of fell over. But she turned around and looked at me and went, “Oh, mom Ah, it's embarrassing. I don't want you to walk us because it's embarrassing when you fall”

For other participants like Tom (CG), internal issues such as the presence of comorbidity reduced PA participation.

… I was walking with Tracey [PwMS] but unfortunately I have arthritis in my hip now. And that comes with age and until I have that rectified, my walking is very limited**.**

##### Breaking out of the cycle of disengagement

As the dyads shared their experiences, it became apparent that the availability of supportive programmes and services or people (health‐care providers and social networks) halted the cycle of disengagement. Accordingly, these resources acted by providing the dyads with the information and tools needed to support learning new skills and action taking with regard to engagement in PA. Once the cycle of disengagement was successfully halted, the dyads described moving towards the cycle of adjustment, where they were able to make changes in order to improve their level of engagement in PA. For some dyads, these changes included planning and making small adjustments to their daily life to ensure that they could participate in PA during the day. For instance, Bob (PwMS) reflected on how he now takes the stairs at work instead of the elevator and how he has been walking over to his co‐workers’ desk rather than emailing them.

#### 3.3.3 Theme 3: Cycle of adjustment

This theme was more apparent among dyads that were currently engaging in some PA and captured their reflections on the adjustments they had to make to incorporate PA into their daily routines. For most of the participants, the process of adjustment occurred over a long period of time, but as soon as they entered into this cycle, they reported needing less support to be able to maintain their PA. For these dyads, the adjustments involved redefining what PA meant to them and shifting their expectations on types of PA to engage in, as well as the mechanism of engagement. This theme was more common in stories shared by dyads who had been living with MS for many years and reflected in two subthemes: (i) acceptance and (ii) innovation and modification.

##### Acceptance

The dyads talked at length about how having MS affected all aspects of their PA experience. They also shared the importance of accepting the reality of MS and adapting to the changes in their abilities to engage in PA, as well as the need to change the way they think about PA.

Linda (PwMS) shared:

…like I mean I'll do as much as I can for the first 2 hours in the morning whether it's cleaning the kitchen or whether it's up and down the stairs with laundry. And that's not every morning. But that's my exercise. And then I'll sit. And if I have to sit in my chair for 2 hours that's okay. And so I just have learned, which was really difficult, just to accept that, so I don't beat myself up about it or try and push myself too much. For me, PA – housework, vacuuming, like it takes me 3 days to vacuum my apartment

##### Innovation and modification

Participants shared their experiences with finding other options or modifying existing types of activities so they could continue being active. Throughout this process, PwMS also reported working collaboratively with their family caregivers to problem‐solving and identify ways to ensure continuous participation in PA.

Leena (PwMS) described how she modified her PA:

So for me, I'm doing things now modified. So I'm still enjoying that I can do those things. Like for aerobics, I can, with the fan blowing on me in my basement, with the treadmill. And I'm holding on because for me the big thing is balance or my legs giving out. So I can still get aerobics on the treadmill.

Jen (PwMS) described working collaboratively with her husband to generate ideas for activities that they could try together:

…I have cognitive difficulties too and I would get lost walking around shopping, which was very difficult to handle in the beginning. But it isn't now. My husband helped me with that. I've got it, there's nothing I can do to change it. But I can work with it and with the support of my husband, coming up with other ideas about things we could do for PA. And that worked amazing.

## 4 DISCUSSION

While there is a growing body of evidence on the experiences of PA participation among PwMS, this literature has been individually oriented and focused primarily on people with mild‐to‐moderate MS‐related disability.^34^, ^42^, ^43^, ^44^, ^45^ Our study is unique because it explores the dyadic perspective of people affected by moderate‐to‐severe MS‐related disability about PA. This has not been done before. Given the literature suggesting the importance of treating the dyad as a unit, this work extends previous findings from populations with other chronic diseases such as cancer, dementia and cardiac disease^27^, ^46^, ^47^, ^48^ to people affected by moderate‐to‐severe MS‐disability.

Across the focus groups, the dyads were concordant in their description of PA as a structured–unstructured continuum. Several activities ranging from aerobic and resistance exercise training to leisure activities and common everyday actions were included in the continuum. The widely varied options for possible PA have been previously reported in survey studies identifying common activities self‐selected by individuals with less severe MS‐related disability.^45^, ^49^ Elsworth et al.^45^ observed that swimming, stretching and walking were the favourite activities selected by 27 PwMS who completed questionnaires as part of a larger focus group study. This evidence was extended by Weikert et al.,^49^ who reported that the most common activities selected by 272 PwMS in a cross‐sectional survey study were walking, weight training and bicycling.

Many participants in our study, especially those individuals with a more severe disability, described engaging in activities in the unstructured end of the continuum. We speculate that this finding may have at least two explanations. First, in clinical practice and research, PA programmes typically focus on exercise training, a subset of PA. Such programmes have traditionally excluded people with higher disability levels.^50^, ^51^ Thus, these individuals may feel that they have no support to engage in the more structured programmes offered in exercise facilities. Second, PwMS were historically told not to engage in PA because of the belief that it can exacerbate the disease process and result in worsening of symptoms especially fatigue.^52^, ^53^ It is possible that some participants in the study still hold some of these beliefs and do not participate in structured exercise to avoid fatigue. This possibility underscores the important role that health‐care professionals have to play in educating people with MS on PA and exercise as a therapeutic strategy for MS management, a finding supported by Learmonth and colleagues in their recent qualitative studies.^54^, ^55^

Some participants also discussed having to move from the structured to the unstructured end of the continuum to enable recovery after experiencing a severe relapse. This finding may be due to the perceived need to reduce the amount of PA participation in order to prevent worsening of the relapse and its symptomatic manifestations, although studies suggest that PA may be safe during a relapse.^52^ This study is the first to show that unstructured and structured PA are not mutually exclusive and that PwMS who have moderate‐to‐severe disability and their family caregivers can go back and forth along the continuum depending on individual circumstances. This finding suggests that a degree of choice and flexibility may be required when designing programmes to support sustained PA participation among those affected by moderate‐to‐severe MS‐related disability. The dyads were also concordant in their beliefs about the benefits of an active lifestyle, corroborating findings from previous individual‐level qualitative research in MS.^33^, ^34^, ^43^, ^44^, ^55^, ^56^, ^57^

The current study has highlighted a novel finding in relation to the influence of mutuality on the dyads’ participation in PA. Participants in this study described PA as an interpersonal experience, with caregivers and care‐recipients sharing very similar struggles, frustrations and adjustments. Considering that PwMS and their family caregivers often react to the impact of the disease as one interdependent unit,^11^ this finding has implications for the design of future interventions. By designing an intervention around equal participation of the dyad in PA, researchers can simultaneously target the health and well‐being of both caregiver and care‐recipient with MS. This approach may be practical in combating the threats that MS and caregiving place on the health of each partner and on them as an interdependent unit.

The dyads in this study also described how the complex interactions between issues related to physical (dis)abilities, loss of independence and freedom, changes associated with MS and negative feedback kept them in the cycle of disengagement and limited the types of PA they could engage in, as well as the mechanism of engagement. This finding highlights the multidimensional nature of PA behaviour, and the crucial roles that physical and psychological factors play in its initiation and maintenance. In clinical practice, a coordinated multidisciplinary health‐care approach targeting the various dimensions of PA may help support sustained behaviour change among individuals affected by moderate‐to‐severe MS‐related disability and those who support them. The need for PA promotion through coordinated health care involving several health professionals has been echoed in previous studies among populations with neurological conditions including MS.^54^, ^56^, ^58^, ^59^, ^60^

The dyads that were able to break out of the cycle of disengagement and enter the cycle of adjustment discussed the importance of supportive resources in the community and within their social networks. These supportive resources were described as the places, programmes and services or people that participants turned to for tools and assistance to support their engagement in PA. Previous studies have repeatedly emphasized the beneficial effect of supportive resources on PA participation among people affected by chronic neurological conditions including MS,^61^, ^62^, ^63^, ^64^ although studies have predominantly focused on the effect of supportive resources on the individual with the disease. In line with Cohen and colleagues^65^ and findings from our study, this body of evidence suggests that strategies such as exposure to peer mentoring support groups at regular intervals and providing advice on practical aspects of engaging in PA may enhance perceptions of social support for dyads affected by moderate‐to‐severe MS‐related disability.

The dyads that were engaging in PA described how they had to accept the reality of MS and work collaboratively together to identify ways of successfully increasing levels of PA. The collaboration described by the dyads also manifested in terms of shared solutions to challenges and emotional support to cope with changing abilities and negative feedback. The positive influence of collaboration within dyads is consistent with previous research evaluating the efficacy of collaborative action on changing dyadic health behaviors.^66^, ^67^, ^68^ For instance, Prestwich et al.^68^ reported that participants who planned PA together with a friend or family member including when and where they would engage in these activities were more physically active than those who planned and acted in isolation. Other researchers have also demonstrated that collaborative adjustment between dyads provides greater perceived control and is associated with better physical health outcomes for the dyad.^25^, ^27^, ^30^, ^69^ Overall, these findings further emphasize the need for researchers and clinicians to situate the focus for intervention development on the needs of the dyad, a practice that has shown beneficial effects in populations with other chronic diseases such as cancer, dementia and cardiac disease.^27^, ^46^, ^47^, ^48^

### 4.1 Limitations

This study has some limitations that warrant consideration. First, our sample comprised majorly of middle‐aged (40‐59 years) and older adults aged ≥60 years. Given the literature suggesting that middle‐aged and older adults with MS are less physically active than young adults affected by the disease,^70^ it is possible that our findings may have been influenced by age‐related factors in addition to MS‐disability. Future researchers may want to explore the differences in PA perspectives between young and older adults affected by moderate‐to‐severe MS‐related disability. Second, we did not have objective information on the PA levels of all our participants. Future researchers may want to gather this information in next studies to provide additional depth of analysis.

Third, although we utilized many strategies to recruit both members of the dyad, recruiting family caregivers proved to be challenging. As a result, a “selection effect” may have occurred where the caregivers who participated in the focus groups may have been different from those who chose not to participate. Fourth, we combined caregivers and PwMS in the same groups rather than having separate groups. While this strategy allowed for an in‐depth exploration of coconstructed experiences, it may have influenced what the partners felt they were able to say during the groups. For example, caregivers may have wanted to protect their care‐recipients by not expressing certain views and vice versa. It is therefore possible that we may have been able to further tease out the nuances of the PA experience between caregivers and PwMS if we had conducted additional separate groups and this would be an interesting avenue for future research.

In addition, it is possible that the amount of caregiving time may influence perspectives on PA. Our study is limited by not having detailed information on caregiving time and tasks. Future researchers in these areas are encouraged to gather this additional information to look at this relationship. Finally, some of the challenges associated with focus groups include domineering or quiet members, the moderator's inability to control the course of discussion and the unnatural setting in which the interviews are conducted.^71^, ^72^ In this study, we attempted to manage these challenges through training of the facilitator, supervision by a senior investigator during each focus group, using a small group composition and ensuring that the focus groups occurred at a place and time of convenience for the participants.

## 5 CONCLUSION

This research has highlighted the need for both people with MS and caregivers to adapt to the impact of the disease on their lives and work together to find options to engage in PA. Given that participants described PA as a continuum of widely varied activities, interventions should not solely focus on promoting structured exercise, but also encourage everyday PA while taking the needs of both caregivers and care‐recipients into consideration. These findings highlight the vital importance of adopting an integrative approach that acknowledges the dyad as a focus for PA interventions, which is highly relevant given the rising burden of MS on society.

## CONFLICT OF INTEREST

The authors declare that there is no conflict of interest regarding the publication of this article.

## References

[1] Motl RW , McAuley E , Sandroff BM , Hubbard EA . Descriptive epidemiology of physical activity rates in multiple sclerosis. Acta Neurol Scand. 2015;131:422‐425. 25598210 10.1111/ane.12352

[2] Kister I , Chamot E , Salter AR , Cutter GR , Bacon TE , Herbert J . Disability in multiple sclerosis a reference for patients and clinicians. Neurology. 2013;80:1018‐1024. 23427319 10.1212/WNL.0b013e3182872855 PMC3653203

[3] Weinshenker BG . The natural history of multiple sclerosis. Neurol Clin. 1995;13:119‐146. 7739500

[4] Myhr KM . Diagnosis and treatment of multiple sclerosis. Acta Neurol Scand Suppl. 2008;188:12‐21. 18439216 10.1111/j.1600-0404.2008.01026.x

[5] O'Hara L , De Souza L , Ide L . The nature of care giving in a community sample of people with multiple sclerosis. Disabil Rehabil. 2004;26:1401‐1410. 15764360 10.1080/09638280400007802

[6] Rivera‐Navarro J , Benito‐Leon J , Oreja‐Guevara C , et al. Burden and health‐related quality of life of Spanish caregivers of persons with multiple sclerosis. Mult scler (Houndmills, Basingstoke, England). 2009;15:1347‐1355. 10.1177/1352458509345917 19797453

[7] Corry M , While A . The needs of carers of people with multiple sclerosis: a literature review. Scand J Caring Sci. 2009;23:569‐588. 19077062 10.1111/j.1471-6712.2008.00645.x

[8] McKeown LP , Porter‐Armstrong AP , Baxter GD . The needs and experiences of caregivers of individuals with multiple sclerosis: a systematic review. Clin Rehabil. 2003;17:234‐248. 12735530 10.1191/0269215503cr618oa

[9] Carton H , Loos R , Pacolet J , Versieck K , Vlietinck R . A quantitative study of unpaid caregiving in multiple sclerosis. Mult Scler. 2000;6:274‐279. 10962548 10.1177/135245850000600409

[10] Murphy N , Confavreux C , Haas J , et al. Economic evaluation of multiple sclerosis in the UK Germany and France. Pharmacoeconomics. 1998;13:607‐622. 17165327 10.2165/00019053-199813050-00013

[11] Pakenham K . Couple coping and adjustment to multiple sclerosis in care receiver‐carer dyads. Fam Relat. 1998;47:269‐277.

[12] Quinn C , Dunbar SB , Clark PC , Strickland OL . Challenges and strategies of dyad research: cardiovascular examples. Appl Nurs Res. 2010;23:e15‐e20. 20420989 10.1016/j.apnr.2008.10.001 PMC2861299

[13] Brush J , Mills K . I Care: A Handbook for Care Partners of People With Dementia. Bloomington, Indiana: BalboaPress; 2014.

[14] Rimmer JH , Marques AC . Physical activity for people with disabilities. Lancet. 2012;380:193‐195. 22818934 10.1016/S0140-6736(12)61028-9

[15] Chodzko‐Zajko WJ . Exercise and physical activity for older adults. Kinesiol Rev. 2014;3:101‐106.

[16] Meneguci J , Sasaki JE , Santos A , Scatena LM , Damião R . Sitting time and quality of life in older adults: a population based study. J Phys Act Health. 2015;12:1513‐1519. 25621767 10.1123/jpah.2014-0233

[17] Motl RW , McAuley E , Snook EM . Physical activity and multiple sclerosis: a meta‐analysis. Mult scler (Houndmills, Basingstoke, England). 2005;11:459‐463. 10.1191/1352458505ms1188oa 16042230

[18] Motl RW , McAuley E , Sandroff BM . Longitudinal change in physical activity and its correlates in relapsing‐remitting multiple sclerosis. Phys Ther. 2013;93:1037‐1048. 23599354 10.2522/ptj.20120479

[19] O'Brien MT . Multiple sclerosis: stressors and coping strategies in spousal caregivers. J Community Health Nurs. 1993;10:123‐135. 8229112 10.1207/s15327655jchn1003_1

[20] Shereman T , Rapport L , Hanks R , et al. Predictors of well‐being among significant others of persons with multiple sclerosis. Mult Scler. 2007;13:239‐249. 10.1177/1352458506070754 17439890

[21] Latimer‐Cheung AE , Pilutti LA , Hicks AL , et al. Effects of exercise training on fitness, mobility, fatigue, and health‐related quality of life among adults with multiple sclerosis: a systematic review to inform guideline development. Arch Phys Med Rehabil. 2013;94:1800‐1828. e1803. 23669008 10.1016/j.apmr.2013.04.020

[22] Cohen ET , Kietrys D , Fogerite SG , et al. A pilot study of feasibility and impact of an 8‐Week integrative yoga program in people with moderate multiple sclerosis‐related disability. Int J MS Care. 2017;19:30‐39. 28243184 10.7224/1537-2073.2015-046 PMC5315321

[23] McAuley E , Motl RW , Morris KS , et al. Enhancing physical activity adherence and well‐being in multiple sclerosis: a randomised controlled trial. Mult scler (Houndmills, Basingstoke, England). 2007;13:652‐659. 10.1177/1352458506072188 17548446

[24] Motl RW , Dlugonski D , Wojcicki TR , McAuley E , Mohr DC . Internet intervention for increasing physical activity in persons with multiple sclerosis. Mult scler (Houndmills, Basingstoke, England). 2011;17:116‐128. 10.1177/1352458510383148 20921239

[25] Berg CA , Upchurch R . A developmental‐contextual model of couples coping with chronic illness across the adult life span. Psychol Bull. 2007;133:920. 17967089 10.1037/0033-2909.133.6.920

[26] Kamen C , Heckler C , Janelsins MC , et al. A dyadic exercise intervention to reduce psychological distress among lesbian, gay, and heterosexual cancer survivors. LGBT Health. 2016;3:57‐64. 10.1089/lgbt.2015.0101 PMC4770846 26652029

[27] Winters‐Stone KM , Lyons KS , Dobek J , et al. Benefits of partnered strength training for prostate cancer survivors and spouses: results from a randomized controlled trial of the exercising together project. J Cancer Surviv. 2015;10(1932‐2267 (Electronic)):1‐12. 26715587 10.1007/s11764-015-0509-0

[28] Prick A‐E , de Lange J , Twisk J , Pot AM . The effects of a multi‐component dyadic intervention on the psychological distress of family caregivers providing care to people with dementia: a randomized controlled trial. Int Psychogeriatr. 2015;27:2031‐2044. 26004290 10.1017/S104161021500071X

[29] Prick A‐E , de Lange J , Scherder E , Twisk J , Pot AM . The effects of a multicomponent dyadic intervention on the mood, behavior, and physical health of people with dementia: a randomized controlled trial. Clin Interv Aging. 2016;11:383. 27099480 10.2147/CIA.S95789 PMC4820235

[30] Lyons KS , Winters‐Stone KM , Bennett JA , Beer TM . The effects of partnered exercise on physical intimacy in couples coping with prostate cancer. Health Psychol. 2016;35:509‐513. 26462060 10.1037/hea0000287 PMC4829498

[31] Dlugonski D , Motl RW , Mohr DC , Sandroff BM . Internet‐delivered behavioral intervention to increase physical activity in persons with multiple sclerosis: sustainability and secondary outcomes. Psychol Health Med. 2012;17:636. 22313192 10.1080/13548506.2011.652640

[32] Pilutti LA , Dlugonski D , Sandroff BM , Klaren R , Motl RW . Randomized controlled trial of a behavioral intervention targeting symptoms and physical activity in multiple sclerosis. Mult Scler. 2014;20:594‐601. 24009162 10.1177/1352458513503391

[33] Kayes NM , McPherson KM , Taylor D , Schluter PJ , Kolt GS . Facilitators and barriers to engagement in physical activity for people with multiple sclerosis: a qualitative investigation. Disabil Rehabil. 2011;33:625‐642. 20695816 10.3109/09638288.2010.505992

[34] Plow MA , Resnik L , Allen SM . Exploring physical activity behaviour of persons with multiple sclerosis: a qualitative pilot study. Disabil Rehabil. 2009;31:1652‐1665. 19479491 10.1080/09638280902738375 PMC4703089

[35] Creswell JW . Research Design: Qualitative, Quantitative, and Mixed Methods Approaches. Thousand Oaks, California: Sage publications; 2013.

[36] Freeman K , O'Dell C , Meola C . Focus group methodology for patients, parents, and siblings. J Pediatr Oncol Nurs. 2001;18:276‐286. 11719908 10.1053/jpon.2001.28455

[37] Duggleby W . What about focus group interaction data? Qual Health Res. 2005;15:832‐840. 15961879 10.1177/1049732304273916

[38] van Eyk H , Baum F . Evaluating health system change‐using focus groups and a developing discussion paper to compile the” voices from the field”. Qual Health Res. 2003;13:281‐286. 12643034 10.1177/1049732302239605

[39] Wilkinson S . Focus groups in health research exploring the meanings of health and illness. J Health Psychol. 1998;3:329‐348. 22021395 10.1177/135910539800300304

[40] Corbin JM , Strauss A . Grounded theory research: procedures, canons, and evaluative criteria. Qual Sociol. 1990;13:3‐21.

[41] Patton MQ . Qualitative Research and Evaluation Methods. Thousand Oaks, California: Sage; 2002.

[42] Kasser S . Exercising with multiple sclerosis: insights into meaning and motivation. Adapt Phys Activ Q. 2009;26(0736‐5829 (Print)):274‐289. 19799098 10.1123/apaq.26.3.274

[43] Dlugonski D , Joyce RJ , Motl RW . Meanings, motivations, and strategies for engaging in physical activity among women with multiple sclerosis. Disabil Rehabil. 2012;34:2148‐2157. 22533641 10.3109/09638288.2012.677935

[44] Borkoles E , Nicholls AR , Bell K , Butterly R , Polman RCJ . The lived experiences of people diagnosed with multiple sclerosis in relation to exercise. Psychol Health. 2008;23:427‐441. 25160577 10.1080/14768320701205309

[45] Elsworth C , Dawes H , Sackley C , et al. A study of perceived facilitators to physical activity in neurological conditions. Int J Ther Rehabil. 2009;16:17.

[46] Smits CHM , de Lange J , Dröes RM , Meiland F , Vernooij‐Dassen M , Pot AM . Effects of combined intervention programmes for people with dementia living at home and their caregivers: a systematic review. Int J Geriatr Psychiatry. 2007;22:1181‐1193. 17457793 10.1002/gps.1805

[47] Martire LM , Schulz R , Helgeson VS , Small BJ , Saghafi EM . Review and meta‐analysis of couple‐oriented interventions for chronic illness. Ann Behav Med. 2010;40:325‐342. 20697859 10.1007/s12160-010-9216-2 PMC4101802

[48] Sher T , Braun L , Domas A , Bellg A , Baucom DH , Houle TT . The partners for life program: a couples approach to cardiac risk reduction. Fam Process. 2014;53:131‐149. 24495204 10.1111/famp.12061 PMC3959575

[49] Weikert M , Dlugonski D , Balantrapu S , Motl RW . Most common types of physical activity self‐selected by people with multiple sclerosis. Int J MS care. 2011;13:16‐20. 24453701 10.7224/1537-2073-13.1.16 PMC3882947

[50] Motl RW . Benefits, safety, and prescription of exercise in persons with multiple sclerosis. Expert Rev Neurother. 2014;14:1429‐1436. 25413175 10.1586/14737175.2014.983904

[51] Toomey E , Coote SB . Physical rehabilitation interventions in nonambulatory people with multiple sclerosis: a systematic review. Int J Rehabil Res. 2012;35:281‐291. 23060086 10.1097/MRR.0b013e32835a241a

[52] Pilutti LA , Platta ME , Motl RW , Latimer‐Cheung AE . The safety of exercise training in multiple sclerosis: a systematic review. J Neurol Sci. 2014;343:3‐7. 24880538 10.1016/j.jns.2014.05.016

[53] Tallner A , Waschbisch A , Wenny I , et al. Multiple sclerosis relapses are not associated with exercise. J Mult Scler. 2012;18:232‐235. 10.1177/1352458511415143 21733890

[54] Learmonth YC , Adamson BC , Balto JM , et al. Multiple sclerosis patients need and want information on exercise promotion from healthcare providers: a qualitative study. Health Expect. 2016;1‐10. https://doi.org/10.1111/hex.12842. 10.1111/hex.12482 PMC5513010 27436592

[55] Learmonth YC , Rice IM , Ostler T , Rice LA , Motl RW . Perspectives on physical activity among people with multiple sclerosis who are wheelchair users: informing the design of future interventions. Int J MS Care. 2015;17:109‐119. 26052256 10.7224/1537-2073.2014-018 PMC4455863

[56] Hale LA , Smith C , Mulligan H , Treharne GJ . “Tell me what you want, what you really really want…”: asking people with multiple sclerosis about enhancing their participation in physical activity. Disabil Rehabil. 2012;34:1887‐1893. 22480377 10.3109/09638288.2012.670037

[57] Learmonth Y , Marshall‐McKenna R , Paul L , Mattison P , Miller L . A qualitative exploration of the impact of a 12‐week group exercise class for those moderately affected with multiple sclerosis. Disabil Rehabil. 2013;35:81‐88. 22656959 10.3109/09638288.2012.688922

[58] Post B , van der Eijk M , Munneke M , Bloem BR . Multidisciplinary care for parkinson's disease: not if, but how! Practi Neurol. 2011;11:58‐61. 10.1136/jnnp.2011.241604 21385961

[59] Thompson JA , Cruickshank TM , Penailillo LE , et al. The effects of multidisciplinary rehabilitation in patients with early‐to‐middle‐stage huntington's disease: a pilot study. Eur J Neurol. 2013;20(1468‐1331 (Electronic)):1325‐1329. 23216520 10.1111/ene.12053

[60] Hass CJ , Okun MS . Time for comprehensive care networks for parkinson's disease. Lancet Neurol. 2010;9:20‐22. 19959399 10.1016/S1474-4422(09)70331-X

[61] Motl RW , Snook EM , McAuley E , Scott JA , Douglass ML . Correlates of physical activity among individuals with multiple sclerosis. Ann Behav Med. 2006;32:154‐161. 16972813 10.1207/s15324796abm3202_13

[62] Damush TM , Plue L , Bakas T , Schmid A , Williams LS . Barriers and facilitators to exercise among stroke survivors. Rehabil Nurs. 2007;32:253‐262. 18065147 10.1002/j.2048-7940.2007.tb00183.x

[63] Ravenek MJ , Schneider MA . Social support for physical activity and perceptions of control in early Parkinson's disease. Disabil Rehabil. 2009;31:1925‐1936. 19479519 10.1080/09638280902850261

[64] Kerstin W , Gabriele B , Richard L . What promotes physical activity after spinal cord injury? an interview study from a patient perspective. Disabil Rehabil. 2006;28:481‐488. 16513581 10.1080/09638280500211932

[65] Cohen S , Gottlieb BH , Underwood LG . Social relationships and health: challenges for measurement and intervention. Adv Mind Body Med. 2001;17:129‐141. 11335207

[66] Prestwich A , Conner M , Lawton R , Bailey W , Litman J , Molyneaux V . Individual and collaborative implementation intentions and the promotion of breast self‐examination. Psychol Health. 2005;20:743‐760.

[67] Wing RR , Jeffery RW . Benefits of recruiting participants with friends and increasing social support for weight loss and maintenance. J Consult Clin Psychol. 1999;67:132. 10028217 10.1037//0022-006x.67.1.132

[68] Prestwich A , Conner MT , Lawton RJ , Ward JK , Ayres K , McEachan RR . Randomized controlled trial of collaborative implementation intentions targeting working adults’ physical activity. Health Psychol. 2012;31:486. 22468716 10.1037/a0027672

[69] Coyne JC , Smith DA . Couples coping with a myocardial infarction: a contextual perspective on wives’ distress. J Pers Soc Psychol. 1991;61:404. 1941511 10.1037//0022-3514.61.3.404

[70] Klaren RE , Sebastiao E , Chiu C‐Y , Kinnett‐Hopkins D , McAuley E , Motl RW . Levels and rates of physical activity in older adults with multiple sclerosis. Aging Dis. 2016;7:1‐7. 27330842 10.14336/AD.2015.1025 PMC4898924

[71] Kidd PS , Parshall MB . Getting the focus and the group: enhancing analytical rigor in focus group research. Qual Health Res. 2000;10:293‐308. 10947477 10.1177/104973200129118453

[72] Morgan DL . The Focus Group Guidebook. Thousand Oaks, California: Sage; 1998.

