# Supplementary material for: Understanding leisure‐time physical activity: Voices of people with MS who have moderate‐to‐severe disability and their family caregivers
Source: Health Expect. 2017 Jul 19;21(1):181–91. doi: 10.1111/hex.12600 (PMC5750693; doi:10.1111/hex.12600)
Supplement: Supplementary file 1 [file HEX-21-181-s001.docx]

**Focus Group Interview Guide**

1. To begin, I would like to know how you define or describe leisure-time physical activity. What does the term mean to you?
2. Would you describe yourself as physically active? Why or why not

Possible probes:

- Typical daily activities (e.g., at work, home, community and for leisure)
- Important activities
- Physical activity related interests and preferences

1. How does your participation in physical activity affect your overall health and well-being?

Possible probes:

- How do you feel when you engage in physical activity?
- Changes in symptoms or impairments?

1. Next, I want you to reflect on your own life and what has encouraged or motivated you to be physically active. In other words, what motivates you engage in physical activity? Let’s create a list.

Possible motivation probes:

- Health
- Appearance
- Enjoyment
- Physical and psychological well-being
- Being with others

1. Based on your knowledge about physical activity, and your previous experiences, what are some of your most significant challenges or issues related to engaging in physical activity? In other words, what discourages you from being physically active? Let’s create another list.

Possible challenges/issue probes:

- Weather (cold/heat/rain)
- Health-related factors
- Safety concerns in the community
- Physical environment (sidewalks/lightning)
- Fear of falls or injuries
- Lack of interest/ motivation
- Lack of someone to do it with
- Lack of time
- Lack of financial resources to engage in physical activity of choice

1. What steps have you taken to manage or reduce these issues in the past?

Possible probes

- Seeking and obtaining information about support resources and services
- Incorporating physical activity into everyday routines (e.g. walking to the photocopier at work, taking stairs instead of elevators)

*Next, I would like to switch to talk about programs, resources and services to support your engagement in leisure-time physical activity*

1. What do you think about the opportunities available in your community for you to engage in physical activity?

Possible probes

- Availability of support resources and services
- Are these resources and services adequate to suit your needs
- Access to information about the support resources available
- Affordability of these resources

1. What are your feelings about having family or friends who live nearby to engage in physical activity together with you?

Possible probes

- The types of shared activities you can engage in,
- The value of having the support of family and friends to engage in leisure-time physical activity

1. Finally, if you could imagine an ideal program that would support you to engage in physical activity, what would it look like?

Possible probes

- A program that you could do alone or with a partner?
- A program that would be delivered at home or at a leisure center (how far would you be willing to travel)
- How many sessions?
- What type of content

Just before we wrap up, is there anything that we have missed or that you have not had a chance to say on this topic?
